# Climate change and gendered vulnerability: A systematic review of women’s health

**DOI:** 10.1177/17455057251323645

**Published:** 2025-03-12

**Authors:** Gulnaz Anjum, Mudassar Aziz

**Affiliations:** 1 Department of Psychology, University of Limerick, Limerick, Ireland; 2 Department of Psychology, University of Oslo, Oslo, Norway

**Keywords:** climate change, gendered vulnerability, women’s health, disproportionate impacts, gender inequalities, mental health challenges, care burden, gender-responsive policies

## Abstract

**Background:**

Climate change is an urgent global threat, with women in low- and middle-income countries (LMICs) disproportionately facing adverse health outcomes. Gendered roles, combined with socioeconomic, cultural, and environmental factors, exacerbate women’s vulnerabilities, increasing the burden of mental health issues, water insecurity, sanitation challenges, and caregiving responsibilities.

**Objectives:**

This review seeks to systematically examine the intersection between climate change and gendered health vulnerabilities, with a particular focus on women. It explores how climate change intensifies gender-specific risks and identifies pathways for integrating gender-responsive policies to mitigate both short- and long-term health impacts.

**Design:**

Following Arksey and O’Malley’s methodological framework, this systematic review mapped key concepts and evidence from studies conducted between January 2011 and January 2024. The review focuses on identifying the multifaceted health impacts of climate change on women, particularly in LMICs and marginalized communities.

**Data Sources and Methods:**

A systematic search was conducted in Web of Science and Scopus databases using key terms and Medical Subject Headings related to climate change, women’s health, gender inequality, mental health, water security, sanitation, and caregiving burdens. Studies were screened and selected based on relevance to the predefined criteria, with data extracted on study design, key findings, and limitations.

**Results:**

From 2163 citations screened, 61 studies were included in the final analysis. The review highlights that climate change disproportionately affects women, exacerbating pre-existing gender inequalities. Specific impacts include heightened mental health challenges, adverse maternal and newborn health outcomes, increased water insecurity, and an intensified caregiving burden. Women in LMICs are particularly vulnerable due to reduced access to resources, healthcare, and decision-making platforms, further limiting their adaptive capacities.

**Conclusion:**

The findings underscore the critical need for gender-responsive climate policies that address both immediate health impacts and the broader socioeconomic and environmental determinants affecting women. Effective climate adaptation strategies must integrate gender perspectives, ensuring that women’s specific vulnerabilities are accounted for in policy frameworks. This review advocates for the empowerment of women through increased access to resources and decision-making, thus enhancing their resilience and adaptive capacity in the face of climate change.

## Plain language summary

How climate change affects women’s health?

This systematic review explores how climate change impacts women’s health. Climate change poses a significant global threat, with women in low- and middle-income countries experiencing heightened health risks, worsened gender inequalities, mental health challenges, and increased caregiving responsibilities. Urgent action is needed through gender-responsive policies, research, and collaborative efforts to address these issues and enhance women’s resilience in climate-induced health challenges. This review aims to contribute to a better understanding of how climate change impacts women’s health and the importance of considering women’s specific needs in climate-related policies and actions.

## Introduction

Climate change is widely recognized as a profound global challenge with far-reaching health, socioeconomic stability, and environmental sustainability implications. Health is conceptualized broadly, encompassing physical health outcomes and mental health, well-being, and the socioeconomic and environmental determinants contributing to health disparities. Among its most concerning impacts is the disproportionate burden it places on women, particularly those in marginalized contexts. These women, often residing in low- and middle-income countries (LMICs), Indigenous communities, and underserved areas within more affluent nations, face heightened vulnerabilities due to socioeconomic, cultural, and environmental factors.^1,2^ This article aims to systematically review the intersection of climate change and gendered health vulnerabilities, emphasizing the urgent need for gender-responsive policies.

The growing body of literature on climate change highlights significant gaps in understanding how environmental changes uniquely affect women. Studies reveal that women’s societal and domestic roles increase their exposure to climate-related risks, adversely impacting their livelihood, financial stability, and physical and mental health.^3^ For instance, severe droughts in Zambia have disrupted women’s livelihoods, affecting their socioeconomic status and reproductive health outcomes. Similarly, climatic fluctuations in Benin correlate with maternal and neonatal health issues, including altered birth weights and increased incidences of low-birth-weight newborns.^3^

The vulnerability of women in marginalized contexts is further compounded by limited access to health services, lower income levels, and reduced participation in decision-making processes. This creates an environment where their ability to respond to climate challenges is significantly hindered.^2^ Exploring the intersection of marginality and gender is crucial because it amplifies and exacerbates existing inequalities, making it more difficult for women to adapt to and mitigate the effects of climate change. In Pakistan, for example, women in informal settlements face increased care burdens due to floods and disease outbreaks, exacerbating existing health and socioeconomic disparities.^4,5^ These compounded vulnerabilities are not merely additive but interact in complex ways that can lead to cascading effects on women’s health and well-being. Women often bear the brunt of caregiving responsibilities, which are intensified during climate crises, further limiting their opportunities for education, employment, and participation in decision-making processes.^6^

Moreover, the existing gender inequalities are exacerbated by the impacts of climate change. Research indicates a significant positive correlation between gender inequality and the prevalence of a dual burden of disease, affecting women disproportionately.^7^ This is illustrated in Bangladesh, where climate change severely limits women’s access to sexual and reproductive healthcare, highlighting the need for a reproductive justice framework.^8^ Addressing these vulnerabilities necessitates a comprehensive review integrating gender perspectives into climate change research and policies. This article provides a systematic review of existing research, identifies existing linkages, and offers an overview for researchers, policymakers, and healthcare providers. By focusing on immediate and long-term health impacts, the article aims to enhance understanding of resilience and adaptive capacity for women facing climate change.

### Conceptual framework

It is important to highlight here that the concept of “vulnerability,” while central to understanding the disproportionate impacts of climate change on women is not without controversy. It can perpetuate stigmatization, stereotyping, and gendered racialization, framing women as inherently weak or passive victims. To address these concerns, this article adopts a feminist and intersectional approach to vulnerability, emphasizing the structural and systemic factors that exacerbate women’s vulnerabilities rather than attributing these to inherent deficiencies. By focusing on the socioeconomic, cultural, and environmental contexts that shape women’s experiences, this framework seeks to avoid reinforcing negative stereotypes. Instead, it highlights the need for gender-responsive policies and interventions that empower women and address the root causes of their increased vulnerability to climate change, thus promoting resilience and equity in the face of global environmental challenges.

In addressing the focus on “gendered vulnerability” within this review, it is crucial to clarify the rationale behind concentrating primarily on women and, in some instances, on “women and children.” The emphasis on women arises from extensive empirical evidence showing that women, especially in LMICs, disproportionately bear the impacts of climate change due to their gendered roles, socioeconomic positions, and systemic inequalities. This focus does not undermine the broader analysis of gender but rather highlights the specific ways in which climate change exacerbates vulnerabilities uniquely affecting women. The inclusion of “women and children” is grounded in the understanding that the well-being of children is inextricably linked to that of their primary caregivers, who are predominantly women. Thus, examining these groups together allows for a more nuanced and comprehensive analysis of how climate change impacts households and communities, reinforcing the interconnected nature of their vulnerabilities.

In addition to a systematic review of the existing literature, we intend to focus on LMICs wherever possible. This was grounded in the recognition that these regions are disproportionately affected by the impacts of climate change. LMICs are particularly vulnerable due to their socioeconomic challenges, including lower income levels, limited access to healthcare, and reduced adaptive capacities. These factors exacerbate the health impacts of climate change, particularly on women who are already marginalized by gender inequalities. The selection of LMICs as a focal point is further justified by the common structural challenges these countries face, such as inadequate healthcare infrastructure, high levels of poverty, and significant dependence on climate-sensitive sectors like agriculture. Despite the diversity within LMICs, this review identifies shared vulnerabilities that make these regions critical for understanding the intersection of climate change and women’s health. Additionally, the article acknowledges the unique regional differences that exist within LMICs, which may influence the manifestation of climate-related health outcomes.

### Current study: rationale and objectives

This article examines the intersection of climate change and gendered health vulnerabilities, with a particular focus on women’s health in marginalized settings. Despite the extensive literature on climate change, there remains a significant gap in understanding its disproportionate impact on women, particularly among LMICs, Indigenous populations, and underserved communities. To address this gap, this review synthesizes recent studies to highlight how climate change exacerbates gender-specific risks, emphasizing the urgent need for gender-responsive policies.

Through a critical analysis of the existing literature, this article aims to map current knowledge and identify gaps in addressing the unique needs of women. The review proposes actionable recommendations for future research, healthcare providers, and international organizations. By advocating for policies that address both immediate health impacts and long-term socioeconomic and cultural factors, the goal is to enhance women’s resilience and adaptive capacity, particularly in marginalized regions. The specific objectives of this article are the following:

- Examine the intersection of climate change and gendered health vulnerabilities, focusing particularly on women’s health.
- Identify the disproportionate impact of climate change on women, especially those in LMICs.
- Highlight the specific health risks faced by women, such as maternal and newborn health, mental health challenges, and increased caregiving burdens.
- Synthesize the existing literature to provide actionable recommendations for gender-responsive climate change policies that address both short-term and long-term health impacts on women.

## Methodology

### Study design

This systematic review was conducted to explore the intersection between climate change and women’s health vulnerabilities, with a focus on identifying the multifaceted impacts and the need for gender-responsive policies. The review followed the methodological framework proposed by Arksey and O’Malley, which is designed to map key concepts and highlight the main sources and types of evidence available on the research topic.^9^

### Search strategy

A systematic search of the literature was performed across Web of Science and SCOPUS, with a strategy carefully developed and tailored for each database to ensure comprehensive coverage of relevant literature. Preliminary searches identified key terms and Medical Subject Headings related to climate change, women’s health, gender inequality, mental health, water security, sanitation, and caregiving burdens. These terms were then refined and applied consistently across both databases. The full search strategy, including the relevant keywords and subject headings, is detailed in Table 1.

**Table 1.** Key words and search strategy.

Keywords	Terms used
Climate change/variability/extremes	(“Climate change” OR “global warming” OR “environmental change” OR “climate variability” OR “climatic variation” OR “extreme weather” OR “climate-related stressors”)
Women’s health	(“Women’s health” OR “female health” OR “maternal health” OR “reproductive health” OR “gender health disparities” OR “sexual health” OR “maternal mortality”)
Gender inequality	(“Gender inequality” OR “gender inequity” OR “gender disparity” OR “gender-based inequality” OR “gender discrimination” OR “gender-based violence” OR “gender roles”)
Mental health	(“Mental health” OR “psychological well-being” OR “psychological health” OR “depression” OR “anxiety” OR “mental illness” OR “psychosocial health” OR “psychological stress”)

Following an initial exploration of each database, the search strategy was refined to ensure the inclusion of appropriate synonyms, topics, and subject headings. The final strategy, designed for a scoping review, was consistently applied across Web of Science and SCOPUS between January 2011 and January 2024. All retrieved studies were exported to EndNote for further analysis, where duplicates were removed. Title and abstract screening was conducted by one reviewer (MA), after which selected references for full-text review were exported into a Microsoft Excel spreadsheet. This spreadsheet was used to organize data extraction, including authors, publication year, study title, location, population demographics, study design, key findings, and limitations.

### Data sources

The search included peer-reviewed journal articles, reviews, and reports published in English. The search strategy was designed to capture a broad spectrum of studies, including quantitative, qualitative, and mixed-methods research, to ensure a thorough exploration of the available evidence. Articles were selected and reviewed based on selection criteria.

### Inclusion and exclusion criteria

The inclusion criteria for this review were.

- Studies published in English.
- Studies focusing on the health impacts of climate change on women.
- Studies addressing socioeconomic, cultural, and environmental factors influencing women’s health in the context of climate change.
- Document type: Article, Early Access, Review Article, Book Chapters

Exclusion criteria included.

- Studies not specifically addressing the intersection of climate change and women’s health.
- Articles focusing solely on men’s health or non-gender-specific impacts of climate change.

### Data extraction and analysis

**Figure 1.**
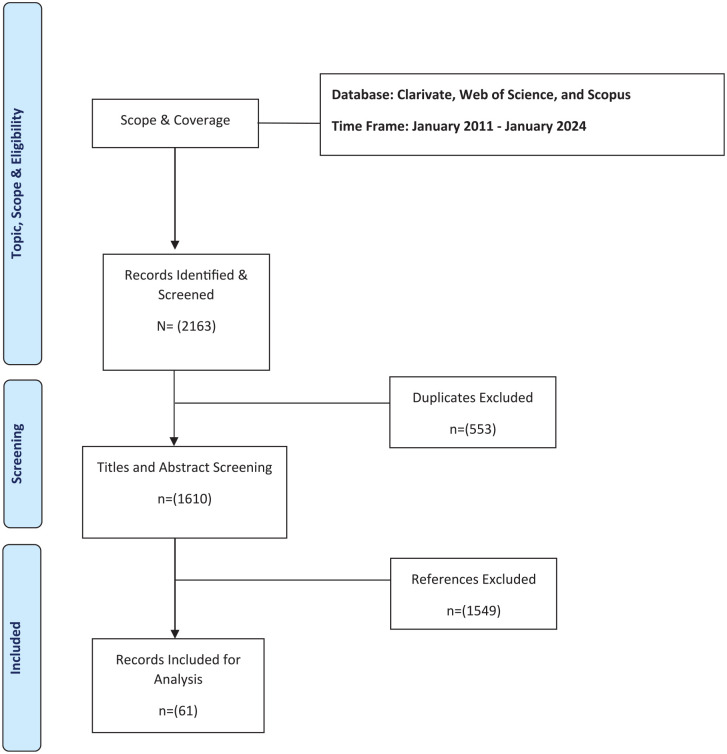
PRISMA diagram of the systematic review process. PRISMA: Preferred Reporting items for Systematic Reviews and Meta-Analyses.

The identified literature was exported to EndNote for further analysis. Duplicates were removed, and the remaining studies were screened for relevance based on titles and abstracts. Key information from the selected studies, including study design, population, outcomes, and key findings, was extracted and organized into thematic categories aligned with the review’s objectives. This article followed guidelines from Page et al.^10^ Details of the process are provided in Figure 1, the Preferred Reporting items for Systematic Reviews and Meta-Analyses (PRISMA) diagram, and a summary table of the included articles in Table 2.

**Table 2.** Summary of the selected articles.

No.	Authors	Title	Source	Year	Key themes
1.	Ylipaa et al.	Climate change adaptation and gender inequality: insights from rural Vietnam	*Sustainability*	2019	Agriculture; climate change adaptation; gender inequality; feminist political ecology; vulnerability; policy; sustainability; Vietnam
2.	Djoudi et al.	Beyond dichotomies: gender and intersecting inequalities in climate change studies	*Ambio*	2016	Adaptation; Climate change; Gender; Intersectionality
3.	Ashrafuzzaman et al.	Exploring gender and climate change nexus, and empowering women in the South Western Coastal Region of Bangladesh for adaptation and mitigation	*Climate*	2022	Gender; climate change and vulnerability; inequality; women empowerment; adaptation; Southwestern coastal region of Bangladesh
4.	Lecoutere et al.	Where women in agri-food systems are at highest climate risk: a methodology for mapping climate-agriculture-gender inequality hotspots	*Frontiers in Sustainable Food Systems*	2023	Climate change; gender inequalities; women; agri-food systems; hotspot mapping; low- and middle-income countries
5.	Lama et al.	Gendered dimensions of migration in relation to climate change	*Climate and Development*	2021	Climate change; climate adaptation; migration; gender dynamics
6.	Phan et al.	Gender inequality and adaptive capacity: the role of social capital on the impacts of climate change in Vietnam	*Sustainability*	2019	Social capital; adaptive capacity; gender inequality
7.	Shayegh and Dasgupta	Climate change, labor availability and the future of gender inequality in South Africa	*Climate and Development*	2024	Gender inequality; labor; wage; skill; Africa; South Africa
8.	Kovaleva et al.	Gender issues within climate change research: a bibliometric analysis	*Climate and Development*	2022	Gender subfield; bibliometric analysis; gender within climate change; gender research; gray literature contribution
9.	Onta and Resurreccion	The role of gender and caste in climate adaptation strategies in Nepal emerging change and persistent inequalities in the far-Western region	*Mountain Research and Development*	2011	Climate change; adaptation; gender; caste; Dalit; Humla; Nepal
10.	Bertana and Blanton	Climate change adaptation, gender, and mainstreaming: the role of gender in Fiji’s relocation initiative	*Climate and Development*	2023	Climate change; adaptation; relocation; gender equality; sea level rise; Fiji; gender mainstreaming
11.	Rainard et al.	Gender equality and climate change mitigation: are women a secret weapon?	*Frontiers in Climate*	2023	Climate change mitigation action; gender mainstreaming; equality; ecofeminism; SDG 13; gender equality
12.	Pinho-Gomes and Woodward	The association between gender equality and climate adaptation across the globe	*BMC Public Health*	2024	Gender inequalities; climate change; climate adaptation
13.	Aryal et al.	Gender and climate change adaptation: a case of Ethiopian farmers	*Natural Resources Forum*	2022	Adaptation; climate change; climate risks; Ethiopia; gender gap
14.	Rai et al.	On the realities of gender inclusion in climate change policies in Nepal	*Policy Design and Practice*	2021	Climate change; climate change policies; gender dynamics; differentiated impacts; women; Nepal
15.	Eastin	Climate change and gender equality in developing states	*World Development*	2018	Climate change; gender equality; women’s rights; development; vulnerability; developing states
16.	Markkanen and Anger-Kraavi	Social impacts of climate change mitigation policies and their implications for inequality	*Climate Policy*	2019	Climate change mitigation policy; just transition; inequality; social impacts; co-impacts
17.	Andrijevic et al.	Overcoming gender inequality for climate resilient development	*Nature Communications*	2020	
18.	Pearse	Gender and climate change	*Wiley Interdisciplinary Reviews-Climate Change*	2017	
19.	Kuehn and McCormick	Heat exposure and maternal health in the face of climate change	*International Journal of Environmental Research and Public Health*	2017	Climate change; maternal health; fetal health; heat exposure
20.	Rylander et al.	Climate change and environmental impacts on maternal and newborn health with focus on Arctic populations	*Global Health Action*	2011	Climate change; environment; maternal and child health; Arctic
21.	Khan et al.	Drinking water salinity and maternal health in coastal Bangladesh: implications of climate change	*Environmental Health Perspectives*	2011	Climate change; hypertension; maternal health; pregnancy; salinity intrusion
22.	Trousdale et al.	Protecting children’s environmental health in a changing climate: a model collaboration of the maternal and child health section and the environment section of APHA	*Maternal and Child Health Journal*	2023	Children; environmental health; climate change; maternal and child health; environmental justice; collaboration
23.	Conway et al.	Climate change, air pollution and maternal and newborn health: an overview of reviews of health outcomes	*Journal of Global Health*	2024	
24.	Roos et al.	Maternal and newborn health risks of climate change: a call for awareness and global action	*Acta Obstetricia et Gynecologica Scandinavica*	2021	Air pollution; climate change; extreme heat; heat wave; maternal health; neonatal health
25.	Hawkinsand Tremblay	Climate change and maternal child health	*MCN—The American Journal of Maternal-Child Nursing*	2023	
26.	Giudice et al.	Climate change, women’s health, and the role of obstetricians and gynecologists in leadership	*International Journal of Gynecology & Obstetrics*	2021	Advocacy; climate change; education; environment; reproduction; women’s health
27.	Chersich et al.	Increasing global temperatures threaten gains in maternal and newborn health in Africa: a review of impacts and an adaptation framework	*International Journal of Gynecology & Obstetrics*	2023	Africa; climate change; heat; maternal and newborn health; pregnancy; reproductive health; temperature
28.	Cil and Cameron	Potential climate change health risks from increases in heat waves: abnormal birth outcomes and adverse maternal health conditions	*Risk Analysis*	2017	Birth outcomes; climate change; heat waves; infant health; maternal health
29.	Rylander et al.	Climate change and the potential effects on maternal and pregnancy outcomes: an assessment of the most vulnerable—the mother, fetus, and newborn child	*Global Health Action*	2013	Climate change; reproductive health; pregnancy outcomes
30.	Yadav and Pacheco	Prebirth effects of climate change on children’s respiratory health	*Current Opinion in Pediatrics*	2023	Air pollution; children; climate change; pregnancy; pulmonary health
31.	Chalupka et al.	Climate and environmental change a generation at risk	*MCN—The American Journal of Maternal-Child Nursing*	2023	Climate change; environmental health; extreme weather; maternal stress; Perinatal health; pregnancy health
32.	Hadley et al.	Impacts of climate change on food security and resulting perinatal health impacts	*Seminars in Perinatology*	2023	Climate change; food security; pregnancy; infant health; pregnancy outcome
33.	Bryson et al.	Seasonality, climate change, and food security during pregnancy among Indigenous and non-Indigenous women in rural Uganda: implications for maternal-infant health	*PLoS One*	2021	
34.	Pardon et al.	Mental health impacts of climate change and extreme weather events on mothers	*European Journal of Psychotraumatology*	2024	Perinatal mental health; extreme weather events; climate change Australian mothers; perinatal mood and anxiety disorders
35.	Kim et al.	What to expect when it gets hotter the impacts of prenatal exposure to extreme temperature on maternal health	*American Journal of Health Economics*	2021	Extreme temperature; climate change; maternal health; pregnancy; hospitalization; health disparity
36.	Walton et al.	Climate shocks and nutrition: the role of food security policies and programs in enhancing maternal and neonatal survival in Niger	*Maternal and Child Nutrition*	2024	Climate change; food security; maternal; neonatal; Niger; nutrition; resilience; subnational
37.	Lokmic-Tomkins et al.	Multilevel interventions as climate change adaptation response to protect maternal and child health: a scoping review protocol	*BMJ Open*	2023	Public health; community child health; qualitative research
38.	Lu	The future of maternal and child health	*Maternal and Child Health Journal*	2019	Future of maternal and child health; life course; technology; innovation; precision; health; big data; climate change; infectious outbreaks; antimicrobial resistance; chronic diseases; infrastructure; social inequality; research; workforce; Health in; all policies
39.	Nakstad et al.	How climate change may threaten progress in neonatal health in the African region	*Neonatology*	2022	Neonates; climate; heat stress; dehydration; breastfeeding; malnutrition; interventions; environment
40.	Stopher et al.	Multiple pathways mediate the effects of climate change on maternal reproductive traits in a red deer population	*Ecology*	2014	*Cervus elaphus*; climate change; demography; fitness; Isle of Rum, Scotland; maternal traits; path analysis; phenology; red deer; reproduction
41.	Mendola and Ha	Beyond the infant in your arms: effects of climate change last for generations	*Fertility and Sterility*	2022	Climate change; temperature; air pollution; reproductive health; intergenerational effects
42.	Stone et al.	Mental health impacts of climate change on women: a scoping review	*Current Environmental Health Reports*	2022	Climate change; gender; women; mental health; scoping study
43.	Rothschild and Haase	Women’s mental health and climate change part II: socioeconomic stresses of climate change and eco-anxiety for women and their children	*International Journal of Gynecology & Obstetrics*	2023	Climate change; climate refugees; depression; eco-anxiety; family planning; gender-based violence; global warming; mental health; natural disasters; perinatal; psychiatry; psychiatry; women
44.	Barkin et al.	Climate change is an emerging threat to perinatal mental health	*Journal of the American Psychiatric Nurses Association*	2024	Perinatal mental health; women’s mental health; mood disorders; postpartum depression; anxiety and anxiety disorders
45.	Chapola et al.	Climate change and its impact on the mental health well-being of Indigenous women in Western cities, Canada	*Journal of Community & Applied Social Psychology*	2024	Climate crisis; climate impacts; Indigenous women’s perspective; mental health well-being; women-led adaptations
46.	Rothschild and Haase	The mental health of women and climate change: direct neuropsychiatric impacts and associated psychological concerns	*International Journal of Gynecology & Obstetrics*	2023	Air pollution; climate change; heat illness; maternal health; mental health; nutritional deficiency; perinatal psychiatry; women
47.	Wright et al.	Climate change and the adverse impact on the health and well-being of women and girls from the Women’s Health Expert Panel of the American Academy of Nursing	*Nursing Outlook*	2023	Climate change; disparities; women and girls; risk factors; effects on health
48.	Sidun and Gibbons	Women, girls, and climate change: Human rights, vulnerabilities, and opportunities	*International Journal of Psychology*	2024	Mental health; climate change; climate consequences; women and girls; gender consequences; women empowerment; exploitation; sustainable development goals
49.	Kadio et al.	Extreme heat, pregnancy and women’s well-being in Burkina Faso: an ethnographical study	*BMJ Global Health*	2024	Qualitative study; maternal health; environmental health
50.	Patel et al.	Climate change and women in South Asia: a review and future policy implications	*World Journal of Science Technology and Sustainable Development*	2020	Women; resilience; climate change; South Asia; extreme events
51.	Fearnley et al.	Environmental correlates of mental health measures for women in Western Australia	*EcoHealth*	2014	Mental health; salinity; environmental health; Bayesian
52.	Bunce et al.	Vulnerability and adaptive capacity of Inuit women to climate change: a case study from Iqaluit, Nunavut	*Natural Hazards*	2016	Climate change; Inuit; women; adaptation; vulnerability; gender; Nunavut
53.	Banerjee et al.	Building capacities of women for climate change adaptation: insights from migrant-sending households in Nepal	*Climatic Change*	2019	Adaptation; climate change; remittances; women; capacity-building interventions
54.	Lee et al.	Women’s health in times of emergency: we must take action	*Journal of Women’s Health*	2021	climate change; women’s health; reproductive health care; social norms; disaster medicine; trauma-informed care
55.	Phuong et al.	Reframing climate change resilience: an intersectional perspective of ethnicity and gender from Vietnam	*Climate*	2023	Climate change and variability; intersectional perspective; livelihood resilience; Vietnam
56.	Dev and Manalo	Gender and adaptive capacity in climate change scholarship of developing countries: a systematic review of literature	*Climate and Development*	2020	Gender; adaptive capacity; women; climate change; developing countries
57.	Sorensen et al.	Climate change and women’s health: impacts and policy directions	*PLoS Medicine*	2018	
58.	Sorensen et al.	Climate change and women’s health: impacts and opportunities in India	*Geohealth*	2021	Ambient air-pollution; pregnancy outcomes; birth-weight; temperature; malaria; exposure; risk; association; stillbirth; mortality
59.	Pandipati and Abel	Anticipated impacts of climate change on women’s health: a background primer	*International Journal of Gynecology & Obstetrics*	2023	Advocacy; children’s health; climate change; climate crisis; climate injustice; women’s empowerment; women’s health
60.	Dickin et al.	Women’s vulnerability to climate-related risks to household water security in Centre-East, Burkina Faso	*Climate and Development*	2021	Drinking water; sanitation; gender; West Africa; adaptation; vulnerability
61.	Tanjeela and Rutherford	The influence of gender relations on women’s involvement and experience in climate change adaptation programs in Bangladesh	*SAGE Open*	2018	Climate change; women; adaptation practices; gender relations

SDG: Sustainable Development Goal; APHA: American Public Health Association.

### Study selection process

Two independent reviewers conducted the study selection process. In the first stage, both reviewers independently screened the titles and abstracts of all retrieved articles based on the predefined inclusion and exclusion criteria. Studies were included if they focused on the intersection of climate change and women’s health vulnerabilities. Additional studies, particularly from LMICs and marginalized communities were searched and included. In the second stage, both reviewers independently reviewed the full texts of the selected studies to confirm their eligibility. Any reviewer discrepancies were resolved through discussion. No automation tools were used during any stage of the study selection process.

### Data items and outcomes

We sought data on several key outcomes related to women’s health in the context of climate change. Specifically, we focused on data on:

- Maternal and newborn health outcomes
- Mental health impacts, including the prevalence of anxiety, depression, and climate-related stress
- Water security and sanitation challenges affecting women’s health, especially in low-income and rural regions
- The caregiving burden on women, particularly how their roles and responsibilities exacerbate their vulnerability to climate stressors

For each study, we aimed to collect all relevant results that aligned with these outcome domains. No assumptions were made when outcomes were unclear or missing, and those studies were excluded from the synthesis.

### Synthesis of findings

The extracted data were synthesized thematically, focusing on the key areas identified: women’s health risks, gender inequality, mental health, water security, sanitation, and caregiving burdens. The review aimed to identify gaps in the current literature and provide actionable recommendations for researchers, policymakers, and stakeholders to develop gender-responsive strategies to address the health impacts of climate change on women.

### Analytical framework and synthesis of findings

The analytical framework underpinning this review is deeply rooted in feminist theory and the concept of intersectionality. This approach recognizes the dynamic interplay of gender, socioeconomic status, cultural norms, and environmental factors in shaping women’s experiences and responses to climate change. By employing this multi-dimensional analytical framework, the review seeks to provide a nuanced understanding of the gendered impacts of climate change and to propose robust, context-specific policy recommendations that can enhance resilience and adaptive capacity among women in marginalized contexts. The synthesis process for data analysis involved integrating the findings from the selected articles to develop a coherent narrative that addresses the objectives of this article. This narrative synthesis was guided by the thematic analysis results and the overarching analytical framework, ensuring that the proposed policy recommendations are grounded in empirical evidence and theoretical insights.

## Results

Initial searches on the databases yielded a total of 2163 citations (see Figure 1), which were exported to EndNote for further analysis. After the removal of duplicates (*n* = 553), title and abstract screening was performed on the remaining unique articles (*n* = 1610). Most of these publications (*n* = 1549) were irrelevant to the topic of the scoping review and were excluded at this stage. Full-text analysis was performed for the remaining publications (*n* = 61). Table 2 lists articles included in this systematic review.

The impact of climate change on women’s health is disproportionate. Women in marginalized contexts are particularly vulnerable due to socioeconomic, cultural, and environmental factors that compound their risks and limit their adaptive capacities. This section synthesizes how climate change affects women’s health compared to men and other genders, highlighting the unique vulnerabilities women face and the broader implications for health equity and climate resilience.

### Gendered vulnerabilities: impact of climate change on women’s health

Climate change disproportionately affects countries like Vietnam and Bangladesh, posing severe risks to agrarian livelihoods, especially for women. Research shows that climate adaptation strategies often neglect the gendered dimensions of vulnerability, exacerbating inequalities and limiting women’s adaptive capacities. Studies highlight that structural constraints and social norms in agriculture and rural settings lead to women’s increased vulnerability to climate impacts, from extreme weather events to long-term environmental changes. Effective adaptation and resilience-building must address these gender inequalities, empowering women through inclusive policies and practices to ensure sustainable agri-food systems and mitigate climate risks.^11–17^

Climate change significantly influences population movement and gendered vulnerabilities, yet the relationship between these factors remains underexplored beyond binary gender comparisons. Research shows that women in developing countries are particularly vulnerable due to physical, socioeconomic, and resource control differences, necessitating a gender-sensitive approach in climate adaptation policies. For instance, a study in a coastal community in Vietnam reveals that gender norms dictate the division of labor and access to social networks, impacting women’s ability to adapt to climate change. Similarly, in South Africa, higher temperatures adversely affect the working hours of low-skilled female labor, particularly in high-exposure sectors, highlighting the need for targeted adaptation policies to mitigate these impacts.

Moreover, bibliometric analyses of climate change literature show a growing recognition of the importance of gender-sensitive perspectives, though much of the research still treats gender in a simplistic male-versus-female dichotomy. A comprehensive review of empirical studies from the Pacific Islands Region underscores the necessity of addressing historical legacies and structural inequalities that contribute to gendered vulnerabilities, advocating for a deeper integration of gender analysis in climate change and security discourse. Effective adaptation strategies must consider the differential impacts of climate change on men and women and empower women by involving them in decision-making processes, thereby building more resilient and equitable climate responses.^11,17–19^

Climate change disproportionately affects women due to their socially constructed roles and responsibilities, which often include caregiving, household management, and subsistence agriculture. These roles increase their exposure to climate-related health risks such as heat stress, waterborne diseases, and malnutrition. For example, women in rural areas often bear the responsibility of fetching water and firewood, tasks that become more arduous and dangerous for their health as climate change exacerbates water scarcity and deforestation.^20^ This increased workload not only affects their physical health but also limits their time and energy for other activities, including education and income-generating work, further entrenching gender inequalities.

Men, while also affected by climate change, often have different roles and responsibilities that can influence their exposure and vulnerability to climate-related health risks. For instance, men engaged in outdoor labor may face increased risks of heat-related illnesses and injuries due to extreme weather conditions.^21^ However, men typically have greater access to resources and decision-making power, which can enhance their adaptive capacity and reduce their overall vulnerability compared to women.^1^ Moreover, the intersectionality of gender with other social categories, such as socioeconomic status, ethnicity, and age, further amplifies women’s vulnerabilities to climate change. Women from low-income households, Indigenous communities, and minority groups often face multiple layers of disadvantage, including limited access to healthcare, education, and economic opportunities. This intersectionality means that climate change impacts are not experienced uniformly across genders, and policies must account for these diverse experiences to be effective.

Despite the increasing focus on climate change adaptation, the gender and cultural dimensions of this process remain underexplored. Studies such as those on Dalit and Lama households in Nepal’s Humla District reveal that shifts in climate patterns, including altered monsoon seasons and decreased snowfall, adversely affect livelihoods, highlighting the intertwined cultural, social, and economic dependencies within these communities. These studies underscore the importance of examining how adaptation processes may exacerbate or alter existing gender inequalities and intercaste dependencies, indicating that cultural and gender dynamics play a critical role in shaping vulnerability and adaptive capacity.^22–26^

The differential impacts of climate change across various geographies and social strata have brought gender to the forefront of international climate discussions. At significant forums like the United Nations Framework Convention on Climate Change Conferences of the Parties, the discourse on gender often centers around gender balance rather than true gender equality. This focus tends to obscure the deeper, intersectional issues of gender inequality perpetuated through climate policy. Research highlights how dominant narratives at these forums are shaped and mobilized, often sidelining more equitable and justice-oriented perspectives. Feminist geographies of climate change advocate for challenging these global conversations to forge new coalitions and techniques aimed at achieving just and equitable outcomes. The increasing recognition of gender-differentiated impacts of climate change has led to gender equality becoming a focal point in international adaptation discourses. However, translating these policy perspectives into local adaptation projects often reveals tensions and inconsistencies. For instance, the relocation of Vunidogoloa in Fiji demonstrates the challenges in aligning the global discourse of gender equality with its practical implementation on the ground. This gap underscores the need for more nuanced and context-specific approaches that address the root causes of gender inequality and ensure that adaptation strategies are inclusive and equitable for all affected communities.^22–26^

Globally, studies have shown a significant correlation between gender equality and improved climate adaptation outcomes. For example, analyses using the Global Gender Gap Index and the Gender Inequality Index reveal that higher gender equality is associated with better environmental performance and climate adaptation readiness. These findings are supported by data from various countries, demonstrating that gender inequalities often increase vulnerability and decrease readiness to adapt to climate impacts. In Ethiopia, research indicates a notable gender gap in climate change adaptation among farming households, largely due to social and cultural barriers that limit women’s capacity to adopt adaptive measures. Addressing these issues requires long-term, gender-informed policies that challenge traditional gender norms and provide equitable opportunities for all.^25–28^

Climate change exacerbates existing gender inequalities, leading to increased vulnerability for women and reinforcing gender disparities across various socioeconomic contexts. Studies show that climate-related stresses, such as extreme weather events and food insecurity, disproportionately affect women, particularly in less-democratic and lower-income states. These adverse impacts on women’s economic independence, health, and social rights often result from preexisting gender disparities in asset ownership and familial responsibilities. The compounded effects of climate change on gender inequality highlight the necessity for climate adaptation policies to integrate gender-sensitive approaches to effectively mitigate these impacts and promote equitable outcomes.^1,29–32^

Moreover, the integration of gender considerations into climate change mitigation policies is crucial to avoid perpetuating or worsening social and economic inequalities. Research indicates that while climate policies can offer co-benefits, they often come with adverse side effects that disproportionately impact vulnerable groups, particularly women. For instance, community forestry in Nepal demonstrates how existing gender inequalities in environmental management can hinder effective adaptation to climate change, emphasizing the need for continuous monitoring and adjustment of gender dynamics within adaptation initiatives. Addressing these issues requires a pro-poor approach, careful planning, and multi-stakeholder engagement to ensure that climate policies enhance, rather than undermine, gender equality. Recognizing and incorporating gender dynamics in climate change research and policymaking is essential for fostering resilient and equitable development in the face of a changing climate.^1,29–32^

### Impact of climate change on maternal and newborn health

Climate change significantly impacts maternal and fetal health, with increasing temperatures and extreme weather events linked to adverse pregnancy outcomes globally. A systematic review of recent literature reveals that temperature extremes can affect birth outcomes such as gestation length, birth weight, and neonatal stress. In addition, environmental factors like air pollution and food security, particularly in sensitive regions like the Arctic, exacerbate these health risks. Studies from Vietnam and coastal Bangladesh, highlight that environmental stressors and saltwater intrusion into drinking water lead to higher incidences of hypertension in pregnancy, further illustrating the complex interplay between climate change and maternal health. This underscores the urgent need for standardized assessment methods and targeted climate adaptation policies focusing on maternal and newborn health.^33–37^

Efforts to address these challenges require interdisciplinary collaboration, as demonstrated by the joint initiatives of the maternal and child health (MCH) and Environment Sections of the American Public Health Association. Such initiatives focus on partnerships between health professionals and climate scientists or community-based initiatives raising awareness about environmental hazards and providing tools for public health professionals to protect vulnerable populations. Despite the existing research gaps, particularly concerning climate-related food and water security and infectious diseases, there is consistent evidence of the detrimental effects of heat and air pollution on birth outcomes. This calls for immediate policy dialogue and action to design climate policies that specifically address the health needs of pregnant women and newborns, ensuring their protection amid worsening climate conditions.^33–37^

Climate change is recognized as a significant global health threat with profound immediate and long-term impacts on vulnerable populations, particularly pregnant women and newborns. Various studies underscore the direct and indirect effects of climate change—such as heat stress, extreme weather events, and air pollution—on maternal and fetal health, highlighting adverse outcomes like preterm birth, gestational diabetes, and stillbirth. These vulnerabilities are exacerbated in poorer countries with limited adaptive capacities, necessitating comprehensive, multisectoral climate adaptation strategies that specifically address the health needs of pregnant women and neonates to enhance societal resilience. The World Health Organization and other international bodies emphasize the urgency of research and health system strengthening to mitigate these effects, advocating for a broad, collaborative approach.^38–42^

Additionally, the intersection of climate change with socioeconomic disparities further intensifies the health risks for disadvantaged communities, including women, children, and minorities. High ambient temperatures and extreme heat waves have been linked to increased rates of maternal and newborn health complications, including hypertensive disorders and infections. This is especially concerning in resource-limited settings where access to healthcare and adaptive infrastructure is inadequate. Proposed adaptation frameworks suggest behavioral changes, health system interventions, and structural modifications to reduce heat exposure and improve health outcomes. A collaborative effort involving robust research, climate financing, and community engagement is crucial for developing and implementing effective climate resilience programs tailored to the needs of pregnant women and newborns, ensuring their health and well-being in the face of a changing climate.^38–42^

Climate change represents a substantial threat to public health in the 21st century, particularly affecting pregnant women, newborns, and children. Recent studies underscore that the impacts of climate change on maternal and perinatal health are both direct and indirect, resulting from environmental disasters such as wildfires, extreme heat, hurricanes, floods, and droughts. These events contribute to a range of adverse outcomes, including gestational complications, pregnancy loss, restricted fetal growth, low birthweight, and preterm birth. Additionally, environmental inequities exacerbate these effects, making socioeconomically disadvantaged populations more vulnerable. The evidence highlights the need for comprehensive, multidisciplinary strategies to mitigate these impacts, including policy reinforcement, increased public and healthcare provider awareness, and enhanced access to quality data and research, particularly in low-resource areas.^43–47^

The literature consistently indicates that climate change not only affects pregnancy outcomes but also has long-term implications for pediatric health, particularly respiratory health. Prenatal exposure to climate-related environmental changes such as increased temperatures, air pollution, and maternal stress is associated with adverse outcomes like impaired lung development, increased prevalence of respiratory infections, and other pulmonary issues. These effects are compounded by the heightened vulnerability of marginalized populations, who face additional challenges such as poverty, food insecurity, and water-related diseases. Addressing these challenges requires a concerted effort from researchers, policymakers, and healthcare providers to develop effective adaptation and mitigation strategies, ensuring the protection and health of the most affected yet least responsible populations. Enhanced antenatal care, targeted interventions, and robust advocacy are crucial to safeguarding MCH in the face of the escalating climate crisis.^43–47^

Climate change poses a significant threat to global health, particularly impacting vulnerable populations such as pregnant women, newborns, and children. Various studies highlight the broad range of mechanisms through which climate and environmental changes affect reproductive health. Direct effects include heat stress and exposure to extreme weather events, while indirect effects arise from increased air pollution and compromised food security. These environmental factors contribute to adverse outcomes such as gestational complications, preterm births, low birth weights, and maternal-infant health issues. The compounded effects of climate change and environmental stressors necessitate comprehensive interventions and multidisciplinary approaches to mitigate their impacts on maternal and perinatal health.^48–53^ The impact of climate change on food security is a critical concern, particularly for pregnant women, who are at an increased risk of nutritional deficiencies and associated health complications. Studies indicate that climate-induced disruptions in food systems exacerbate malnutrition, leading to obstetric complications and increased maternal and infant mortality rates. Indigenous communities and socioeconomically disadvantaged groups are particularly vulnerable due to existing inequities and heightened sensitivity to environmental changes. Moreover, the perinatal period is marked by heightened susceptibility to mental health disorders, with extreme weather events exacerbating stress, anxiety, and trauma among new mothers. Research underscores the importance of addressing the mental health impacts of climate change on the perinatal population, advocating for targeted interventions and policies to protect maternal mental health and well-being in the face of increasing environmental challenges. These findings call for urgent and coordinated global efforts to enhance adaptive capacities and resilience among the most affected populations, ensuring sustainable health outcomes for future generations.^48–53^

Climate change is increasingly recognized as a significant threat to maternal and neonatal health, particularly in low-resource settings like Niger and across Africa. The frequent environmental crises, including recurrent droughts and floods, have led to chronic food insecurity, adversely affecting maternal and neonatal nutrition. Studies have shown that while policies and programs targeting food security and nutrition have been implemented, their impact has been hindered by poor financial resources and suboptimal execution. The Nigerien government’s multisectoral approach to improving food security has made some progress in vulnerable regions, but more needs to be done to enhance resilience, especially through targeted investments in health, food, agriculture, education systems, and social protection.^54–59^

The broader impacts of climate change on MCH are multifaceted, involving direct and indirect effects from extreme weather events, heat stress, and air pollution. These environmental changes disrupt food security, exacerbate malnutrition, and increase the burden of disease, particularly in Indigenous and socioeconomically disadvantaged communities. Studies emphasize the need for sustainable, culturally appropriate interventions to mitigate these impacts. Effective strategies include health education, nature-based solutions, improved housing quality, and enhanced healthcare infrastructure. Additionally, there is a growing recognition of the mental health impacts of climate change on perinatal populations, with extreme weather events linked to increased stress, anxiety, and trauma among new mothers. Addressing these challenges requires a comprehensive approach that integrates policy efforts, research, and community-based interventions to protect the health and well-being of mothers and infants in the face of climate change.^54–59^ Research highlights the intergenerational effects of climate change, where poor maternal health due to environmental stressors leads to adverse reproductive outcomes in offspring, perpetuating a cycle of vulnerability. As climate conditions deteriorate, the health of both parents and their children is compromised, emphasizing the need for long-term strategies that extend beyond immediate health interventions. Strengthening policies to reduce emissions, enhancing healthcare providers’ roles as advocates, and ensuring equitable access to resources are critical to mitigating the long-term impacts of climate change on maternal and neonatal health. Collaborative, transdisciplinary efforts and robust research are essential to developing effective solutions and guiding policy to address the pressing health threats posed by a changing climate.^54–59^

### Mental health

The escalating levels of atmospheric greenhouse gases due to human activities have led to a global increase in surface temperature, resulting in severe environmental and health consequences. The adverse outcomes of climate change include extreme weather events, compromised food, water, and air quality, decreased food security, and a rise in vector-borne diseases. These impacts further exacerbate political and economic instability and lead to mass migration, reducing access to healthcare resources. Women, particularly pregnant women, are disproportionately affected by these changes, facing higher risks of heat and particulate-related morbidity and mortality, pregnancy complications, and mental health issues. Healthcare providers play a crucial role in preparing for these challenges through political advocacy, family planning services, and focused nutrition and lifestyle counseling.^44,60–63^

Climate change also disrupts the timing of menarche, with significant health implications for women. Environmental factors influenced by climate change, such as increased frequency of hurricanes and extreme weather events, can alter the age of menarche, leading to increased disease burden in areas like mental health, fertility, cardiovascular disease, and bone health. The mental health impacts of climate change on women, including increased gender-based violence, care responsibilities, and eco-anxiety, are profound and understudied. Women, especially women of color, are more susceptible to adverse mental health outcomes due to socioeconomic stressors and displacement caused by climate-related events. Addressing these issues requires climate policies that reflect women’s unique needs and proactive involvement from healthcare practitioners in climate-related advocacy to ensure the health and safety of their patients.^44,60–63^

Climate change poses a significant and emerging threat to perinatal mental health, as extreme weather events and environmental degradation exacerbate existing challenges faced by pregnant and postpartum women. Various factors such as role adjustments, fluctuating hormones, and the trauma of natural disasters contribute to negative mood symptoms and mental health issues during the perinatal period. Despite the increasing prevalence of terms like “eco-anxiety” and “climate despair,” there is a notable gap in discussions about how the climate crisis specifically impacts maternal mental health, which is crucial given the broader implications for family units and overall well-being.^48,61,64–66^

The impact of climate change on women’s mental health extends beyond the perinatal period, affecting women and girls through various direct and indirect pathways. Environmental stressors such as heat, air pollution, food insecurity, and infectious diseases increase risks for depression, post-traumatic stress disorder (PTSD), and other neuropsychiatric symptoms. Indigenous women in Western Canada face compounded mental health disparities due to the intersection of environmental degradation and ongoing colonial impacts. Climate change also threatens women’s general and reproductive health, leading to issues such as displacement, interrupted education, and increased gender-based violence. Empowering women and adopting gender-sensitive climate policies are crucial for mitigating these adverse effects and advancing human rights and sustainable development goals. Overall, a comprehensive approach involving policy interventions, community-led initiatives, and increased research is essential to address the multifaceted challenges climate change poses to women’s mental health.^48,61,64–66^

Climate change disproportionately impacts women, particularly in regions where gender inequality is prevalent, exacerbating existing socioeconomic and health disparities. Studies from Bangladesh, Burkina Faso, South Asia, and the Arctic highlight how extreme weather events, rising temperatures, and other climate-related changes intensify challenges for women. In Bangladesh, climate events further restrict women’s access to sexual and reproductive health services, heightening their vulnerability to physical, mental, and psychological harm. Similarly, in Burkina Faso, extreme heat negatively affects women’s physical and mental health, their ability to care for themselves and their children, and their economic activities, underscoring the need for public health initiatives and better communication from health professionals about heat risks.^8,67–70^

Research from South Asia emphasizes that climate change significantly impacts women’s socioeconomic conditions, particularly in agriculture, food security, and health, calling for gender-sensitive policies and intervention-based research to build resilience. In Western Australia, environmental degradation, such as soil salinization, has been linked to mental health issues, although individual-level studies showed no direct associations, suggesting the complexity of these interactions. In the Arctic, Inuit women experience unique vulnerabilities due to climate change, affecting traditional activities like berry picking and sewing, with adaptive capacities influenced by mental and physical health, education, and access to resources. These studies collectively demonstrate the critical need for integrating gender perspectives in climate adaptation policies to enhance resilience and address the specific needs of women in diverse contexts.^8,67–70^

### Water and food security, and sanitation

Climate change exacerbates vulnerabilities in water and sanitation services, disproportionately affecting women due to their gendered roles and responsibilities. In regions like the Centre-East of Burkina Faso, variable climate conditions such as extended dry spells and intense rainfall events disrupt household water security, impacting health, well-being, and livelihoods. Women’s vulnerabilities are heightened by social and cultural norms, including those influenced by ethnicity, which dictate water usage and access. These findings underscore the necessity of developing climate-resilient water and sanitation services that account for social drivers of vulnerability, ensuring that gender-specific needs and capacities are addressed in adaptation strategies.^71–73^

Health impacts from climate change, including heat exposure, poor air quality, and waterborne diseases, affect men and women differently based on geographic and socioeconomic factors. Women in LMICs face greater risks due to existing gender-based health disparities, which climate change is likely to exacerbate. Integrating a gendered perspective into climate, development, and disaster-risk reduction policies is crucial. This requires better data collection, monitoring of gender-specific targets, and equitable stakeholder engagement. Empowering women as educators, caregivers, and agents of social change can enhance the effectiveness of mitigation and adaptation policies. In Bangladesh, women’s critical roles in ensuring food security, water management, and livelihoods highlight their essential contributions to climate adaptation. However, significant challenges remain in incorporating women as active agents in climate adaptation programs due to entrenched gender power dynamics. Addressing these challenges is key to leveraging women’s potential in building resilience against climate impacts.^71–73^

Climate change poses significant health threats globally, with a particularly pronounced impact on women, especially those in low-income and disadvantaged communities. Increased levels of greenhouse gases have led to a rise in global temperatures, resulting in extreme weather events, poor air and water quality, and reduced food security. These changes exacerbate vulnerabilities for women, including increased risks of heat-related illnesses, reproductive health complications, and mental health issues. The impacts are further compounded by social and economic disparities, limiting women’s access to essential health services and resources. Addressing these challenges requires integrating gender perspectives into climate policies, improving data collection on gender-specific impacts, and empowering women as key agents in climate adaptation and mitigation efforts.^21,40,44^

Research highlights the need for climate-resilient strategies that consider the unique vulnerabilities and capacities of women. For instance, in regions like Burkina Faso and Bangladesh, women’s roles in water management and food security are critical yet hindered by gender norms and limited resources. Similarly, in India, rapid environmental changes threaten to widen existing gender-based health disparities. Effective climate adaptation requires multisectoral coordination and the inclusion of women in decision-making processes. Empowering women through education, advocacy, and equitable policy frameworks can enhance their ability to adapt to and mitigate climate impacts, ultimately contributing to more resilient and equitable societies.^21,40,44^

### Social norms and exacerbation of women’s care burden

The studies collectively highlight the critical intersection of gender and climate change, emphasizing how climate-induced stressors disproportionately affect women, especially in developing regions. The research conducted in Vietnam’s Nam Dong District reveals significant disparities in resilience levels among different ethnic and gender groups, with women, the poor, and ethnic minorities exhibiting lower resilience. This underscores the necessity for targeted policies that enhance financial, human, and social capitals, tailored to the cultural and social contexts of these vulnerable groups. The study recommends comprehensive training programs and the strengthening of institutional systems to improve overall household resilience, particularly for disadvantaged groups.^13,74,75^

Further reinforcing these findings, a systematic review of literature from developing countries shows that gender, alongside social factors, significantly influences people’s adaptive capacities to climate crises. In agriculture-dominated economies, women’s adaptive capacities are deeply affected by social norms, power dynamics, and control over assets. The study in Bangladesh’s Southwestern Coastal Region provides a case in point, demonstrating that climate vulnerability exacerbates gender inequality, making women more susceptible to both immediate and long-term climate impacts such as natural disasters and salinity intrusion. To mitigate these effects, the study advocates for empowering women through education, economic opportunities, and participation in adaptation efforts. Implementing gender-sensitive policies and promoting women’s roles in climate adaptation are essential for building resilient communities and addressing the socioeconomic challenges posed by climate change.^13,74,75^

The studies collectively emphasize the gendered vulnerabilities and adaptive capacities of women during times of crisis, particularly focusing on climate change and its associated impacts. Women historically face gender-specific disadvantages during emergencies, which are exacerbated by restricted access to healthcare, economic instability, and conservative social norms. These inequities are evident during both acute crises, such as natural disasters and pandemics, and longer-term emergencies like climate change. Women’s health is particularly at risk due to limited access to maternal and reproductive health services during these times. Additionally, women are over-represented in low-wage jobs and unpaid caregiving roles, which further limit their financial and economic stability. The studies highlight the increased vulnerability to violence and the limited pathways to escape trauma during emergencies, emphasizing the need for trauma-informed care, more women in leadership roles, educational initiatives, and advocacy from health professionals to protect and advance women’s health.^71,76,77^

Specifically, the research in Burkina Faso, Nepal, and Bangladesh reveals how climate change exacerbates gender disparities in water security, adaptive capacities, and livelihood resilience. In Burkina Faso, women’s vulnerabilities to water insecurity are driven by gendered roles and norms, with ethnic differences also playing a role. In Nepal, capacity-building interventions for women left behind by migrating men have shown to enhance adaptive capacities and flood preparedness. In Bangladesh, post-cyclone Aila, women’s absence of male household members has led to innovative adaptation strategies leveraging social capital and local knowledge. These findings challenge the perception of women as passive victims of climate change and highlight their active agency in adaptation. The studies collectively underscore the importance of gender-sensitive policies and programs that address the social drivers of vulnerability and empower women to build resilience against climate change and other emergencies.^71,76,77^

Climate change exacerbates socioeconomic vulnerabilities, particularly among women in developing countries such as Bangladesh and India, due to their significant roles in food security, water management, and livelihood sustenance. Women in these regions are disproportionately affected by climate change-induced disasters, resulting in heightened risks to their health, economic stability, and overall well-being. These impacts are compounded by pre-existing gender inequalities, limited access to resources, and exclusion from decision-making processes. Despite these challenges, women play a crucial role in climate adaptation, utilizing their knowledge and experience to develop effective strategies for coping with environmental changes. Empowering women through education, economic support, and inclusion in policy frameworks is essential to enhance their adaptive capacities and mitigate the adverse effects of climate change.^21,72,73,78^

The studies highlight the necessity of integrating gender perspectives into climate, development, and disaster-risk reduction policies to address the differential impacts of climate change on men and women. Gender-based health disparities, influenced by socioeconomic, cultural, and physiological factors, can be mitigated through targeted policy actions and multi-sectoral coordination. Empowering women as agents of social change can significantly improve mitigation and adaptation interventions, reducing negative health outcomes and enhancing community resilience. The literature emphasizes that while women are vulnerable to climate change, they are also proactive in adaptation efforts, advocating for environmental restoration and sustainable practices. Bridging knowledge gaps and implementing inclusive strategies are critical to minimizing women’s vulnerability to climate change and ensuring sustainable development.^21,72,73,78^

## Discussion

Climate change disproportionately affects women due to socioeconomic, cultural, and environmental factors. Women’s caregiving roles heighten their exposure to health risks like heat stress, malnutrition, and waterborne diseases, compounded by systemic gender inequalities that limit their adaptive capacity.^20^ Additionally, non-binary and transgender individuals face unique challenges, including social stigma and discrimination, further limiting their access to essential services during climate disasters.^79^ Inclusive climate policies addressing these vulnerabilities are crucial for equitable health outcomes.

In global health, climate change significantly impacts maternal and newborn health, with pregnant women and newborns particularly vulnerable to heat stress, extreme weather, and air pollution.^42^ In Africa, neonates face heightened risks due to climate change-induced conditions, necessitating interventions such as health education and improvements in housing and food systems.^57^ The intersection of climate change with maternal and neonatal health in Africa, Asia, and South America presents critical challenges, with escalating heatwaves in these regions increasing risks of preterm births, low birth weight, and stillbirths.^80^ This underscores the urgency of integrating climate change into healthcare planning and intervention to mitigate its compounded impacts on vulnerable populations.^57^ Climate change significantly impacts mental health and developmental outcomes. Prenatal stress and environmental degradation can lead to adverse pregnancy outcomes and alter children’s developmental trajectories, necessitating predictive tools and interventions in vulnerable communities.^81^ Rising temperatures and extreme heat negatively affect maternal and neonatal health, highlighting the need for further research and uniform assessment standards.^35^ In the United States, climate change-related exposures, such as extreme temperatures and air pollution, are linked to adverse perinatal and maternal outcomes, emphasizing the importance of addressing climate change to protect maternal and neonatal health.^79^ Obstetricians and gynecologists are crucial in combating climate change impacts by raising awareness, educating, and advocating for mitigation strategies.^40^ Additionally, prenatal stress from factors like malnutrition and inflammation is linked to poor birth outcomes and behavioral and psychological diagnoses in children, highlighting the need for targeted interventions, particularly in low-resource settings.^82^ Parental environmental factors, including stress, significantly impact offspring’s long-term health, increasing risks of chronic diseases such as cardiovascular, metabolic, immune, and neurological conditions. This underscores the importance of considering both maternal and paternal environmental exposures and lifestyle choices in prenatal care.^83^ Prenatal stress, including smoking, alcohol abuse, and drug use, can harm fetal development, necessitating comprehensive prenatal care and public health campaigns to reduce these risks. The placenta plays a crucial role in mediating the effects of maternal mood on fetal and child development, indicating a complex interplay between maternal psychological and physiological processes.^84^ Stress exposure in parents before and during pregnancy can influence offspring brain development through epigenetic mechanisms, highlighting the biological basis for the intergenerational transmission of stress effects.^85^ Emotional and psychological states during pregnancy significantly affect children’s emotional and cognitive outcomes, emphasizing the importance of prenatal mental health.^86^

Climate change exacerbates preexisting gender inequalities, imposing additional economic and social constraints on women, particularly in developing regions, hindering their economic independence and well-being.^1^ Women bear disproportionate costs of climate change, such as asset inequalities, increased familial burdens, and disaster exposure, which diminish their economic and social rights, especially in less democratic and economically underdeveloped states. A gendered perspective in climate, development, and disaster-risk policies is essential to mitigate health problems, protect sexual and reproductive rights, and empower women as agents of social change. This perspective also addresses the undervaluation of women’s contributions to sustainable development and climate strategies.^87^

The exploration of gender inequality in Vietnam offers a valuable case study, highlighting how women’s limited access to decision-making power and resources within these networks constrains their ability to effectively adapt to climate change. These gendered constraints not only affect individual women but also weaken community-wide resilience by sidelining the knowledge and capacities women could contribute to climate adaptation strategies.^17^ Addressing gender inequalities is crucial for climate-resilient development, with improvements possible under sustainable development scenarios.^29^ Gender analysis in climate research is essential, as gender relations drive social transformations related to climate change, challenging gender-blind approaches.^32^ In Bangladesh, gender inequalities exacerbate women’s vulnerability to climate impacts, highlighting the need for gender-sensitive policies.^88^ The integration of gender perspectives into climate policies requires innovative, equitable strategies.^89^ For example, women in Pakistan’s informal settlements face increased care burdens due to climate-related floods.^4^ Gender inequalities in LMICs are both reflected and intensified by climate change, with studies showing correlations between gender inequality and health issues, such as in Zambia and Benin, where climate fluctuations affect maternal and neonatal health.^3^ Women’s disproportionate vulnerability due to social status and working conditions emphasizes the need for their inclusion in decision-making to reduce climate impacts and promote gender mainstreaming.^90^

Maternal and newborn health are particularly vulnerable to climate change, with heightened risks from extreme heat and pollution, leading to adverse outcomes like preterm births.^80^ Gender-sensitive healthcare and policies are essential to support maternal and newborn health.^91^ Women in the Global South face severe mental health impacts from climate change, such as depression and PTSD, requiring integrated mental health services in adaptation plans to build resilience.^61^ Climate change disproportionately affects women, particularly in water security and sanitation. Women’s roles in water collection and sanitation management place them at increased risk of health issues like waterborne diseases and psychosocial stress.^92,93^ In regions like Burkina Faso and rural India, gender norms exacerbate these vulnerabilities, underscoring the need to include women’s voices in water and sanitation projects.^20,71^ Climate change also exacerbates women’s caregiving burdens, limiting their opportunities for education and employment, thus entrenching gender inequalities and necessitating targeted policy interventions.^94,95^

The mental health of women in the Global South is notably affected by climate change. Studies highlight how climate risks and extreme weather exacerbate mental health challenges, such as depression, PTSD, and other neuropsychiatric symptoms.^61^ This research emphasizes the need for gender-sensitive mental health services integrated into climate adaptation plans.^91^ Additionally, the impact of climate change on women’s health in India highlights the importance of incorporating a gendered perspective into policy frameworks to mitigate adverse outcomes and empower women as agents of social change.^72^

Water scarcity and sanitation challenges due to climate change have severe implications for women’s health and well-being. Studies from Burkina Faso and India reveal how gender norms and inadequate infrastructure increase women’s vulnerabilities, emphasizing the need for gender-sensitive, climate-resilient water and sanitation services.^20,71^ In Bangladesh and Kenya, inadequate water access leads to increased health burdens, environmental pollution, and reduced economic productivity, highlighting the importance of tailored adaptive practices and improved sanitation facilities.^93,96^ Women and children are particularly vulnerable to climate change-induced diseases, with temperature variability increasing risks from vector- and water-borne diseases. Climate change poses significant health threats to pregnant women and newborns, highlighting the need for comprehensive research and strengthened health systems.^42^ In Bihar, India, erratic rainfall and temperature fluctuations increase disease susceptibility among women and children, necessitating public awareness and targeted health interventions. Urban health is also at risk due to climate variability and disaster risk, requiring preventive measures and public health education.^97^ Among Ethiopia’s Afar pastoralists, gender inequality exacerbates women’s vulnerability to food insecurity and climate-related risks, underscoring the need for gender-sensitive adaptation strategies.^98^ In Arunachal Pradesh, India, Adi farmers’ adaptation strategies reveal gender-specific impacts of climate change, influenced by socioeconomic status.^99^ These studies collectively stress the need for gender-sensitive approaches in climate adaptation and policymaking to address the distinct challenges women and children face, particularly regarding water scarcity, sanitation, and health vulnerabilities.

The critical review of the selected literature in this article reveals significant disciplinary differences and methodological approaches that shape our understanding of the intersection between climate change and women’s health. Quantitative studies, predominantly from public health and environmental science, provide statistical data on health impacts such as heat-related illnesses and maternal health complications, yet often lack the sociocultural context that qualitative research from sociology and gender studies offers. Qualitative studies provide rich insights into how cultural norms and socioeconomic factors exacerbate women’s vulnerabilities but are limited by smaller sample sizes and challenges in generalizability. Mixed-methods studies attempt to integrate these strengths, offering a more comprehensive view, though they sometimes struggle with consistency in findings. This review highlights the need for more interdisciplinary research that bridges empirical data with contextual analysis, ensuring that gender-responsive policies are both data-driven and contextually informed. Building on these findings, the following recommendations are proposed to enhance gender-sensitive approaches to climate resilience.

In contrast to previous research that often focuses on high-income countries, this review highlights critical gaps in gender-responsive policy development in LMICs, emphasizing the need for more localized, context-specific interventions. This strengthens the call for urgent integration of gender perspectives into climate adaptation and mitigation efforts, a recommendation consistently echoed in the broader literature. Our findings underscore the necessity for policymakers to address immediate health impacts and the long-term socioeconomic and cultural factors that reinforce gendered health vulnerabilities.

### Limitations

While this review provides a comprehensive analysis of the intersection between climate change and women’s health, it is important to acknowledge several limitations that may affect the generalizability and interpretation of the findings. First, the systematic review process, despite being rigorous, is inherently constrained by the availability and quality of the existing literature. The studies included in this review vary widely in terms of their methodological approaches, sample sizes, and regional focuses, which can lead to challenges in synthesizing findings and drawing definitive conclusions. Additionally, the inclusion of studies from diverse socioeconomic and cultural contexts, particularly across LMICs, presents the risk of oversimplifying complex, context-specific issues. While we have endeavored to highlight both commonalities and differences across regions, the heterogeneity of the data limits the extent to which findings can be universally applied.

Moreover, the potential for publication bias cannot be overlooked, as studies with significant or positive results are more likely to be published, which may skew the overall understanding of the impact of climate change on women’s health. Another limitation is related to the generalizability of the findings; while this review focuses on LMICs, the diversity within these regions means that not all conclusions may be applicable to every context. Further research is needed to explore the nuances of climate change impacts on women’s health in specific local settings, particularly through longitudinal and mixed-methods studies that can capture the dynamic and evolving nature of these impacts. Finally, while this review attempts to incorporate an intersectional perspective, the complexity of intersecting social categories such as gender, race, and socioeconomic status may not be fully captured in the existing literature, indicating a need for more comprehensive, intersectional research in this area. Moreover, future research could more fully incorporate intersectional analyses (e.g., through disaggregated data collection by gender, race, class, and other identities).

### Recommendations and the way forward

These studies underscore the urgent need for gender equity in climate action, advocating for policies involving women in all climate adaptation and mitigation stages. A gender-responsive approach is crucial, recognizing women’s vulnerabilities and their gender roles as key actors in climate resilience. This includes promoting gender-sensitive healthcare, enhancing women’s access to resources and decision-making, and implementing targeted interventions to mitigate health impacts, thus ensuring that marginalized groups are not left behind.^100,101^ Despite recognizing women’s roles in climate action, challenges remain in effectively integrating gender equality into climate policies. Prevailing gender assumptions and limited gender-sensitive approaches hinder progress, necessitating a more informed pursuit of gender equality in climate policy.^102^ Gender-sensitive strategies are essential in addressing the psychological and health burdens women face, particularly in conflict zones and climate-affected areas, where they bear disproportionate care and labor burdens.^5,94^

The intersection of climate change, gender inequality, and labor burdens highlights the need for systemic approaches to resilience. Climate-induced shocks, especially in agriculture-dependent and economically underdeveloped states, exacerbate gender disparities, undermining women’s economic and social rights.^1^ Gender-sensitive policies must address these issues comprehensively to ensure sustainable development and welfare outcomes.^95^

Moreover, climate change’s psychological toll on women, particularly in conflict zones, demands systematic mental health support. The complex, prolonged nature of trauma in these contexts requires sustainable mental health interventions that acknowledge the unique pressures faced by women.^103,104^ Addressing these challenges requires integrating gender considerations into climate policies, investments, and interventions to mitigate adverse health and social outcomes.

## Conclusion

This systematic review shows the critical nexus between climate change and women’s health, highlighting the compounded vulnerabilities women face due to intersecting socioeconomic, cultural, and environmental determinants. The findings indicate that climate change intensifies pre-existing gender inequalities, profoundly affecting women’s physical and mental health, amplifying their caregiving responsibilities, and compromising their access to vital resources such as water and sanitation. These impacts are particularly acute in LMICs, where women bear a disproportionate burden of the adverse effects of extreme weather events, food insecurity, and health disparities. The review advocates for incorporating gender-responsive frameworks within climate adaptation and mitigation strategies, emphasizing the imperative for policies addressing women’s immediate health ramifications. We advocate for women’s empowerment by enhancing their access to resources, increasing their involvement in decision-making processes, and implementing targeted interventions that bolster their resilience. Addressing these complex challenges necessitates a comprehensive approach integrating gender considerations into global and local climate policies. This ensures that women’s health and well-being are systematically prioritized within the broader context of climate change adaptation and mitigation efforts. Failure to integrate gender into climate policies will not only exacerbate existing inequalities but also undermine global efforts to build resilient communities.

## Declarations

**Ethics approval and consent to participate:** The research conducted here is review research that does not include interaction with participants. Therefore, ethics approval is not required.

**Consent for publication:** Our research does not use any copyrighted material, so we do not need consent for publication.

**Availability of data and materials:** This is a review article based on existing research. No additional data and materials are attached to this review article; reviewed documents are listed in the article.
